# First *de novo KCND3* mutation causes severe Kv4.3 channel dysfunction leading to early onset cerebellar ataxia, intellectual disability, oral apraxia and epilepsy

**DOI:** 10.1186/s12881-015-0200-3

**Published:** 2015-07-21

**Authors:** Katrien Smets, Anna Duarri, Tine Deconinck, Berten Ceulemans, Bart P. van de Warrenburg, Stephan Züchner, Michael Anthony Gonzalez, Rebecca Schüle, Matthis Synofzik, Nathalie Van der Aa, Peter De Jonghe, Dineke S. Verbeek, Jonathan Baets

**Affiliations:** Neurogenetics Group, VIB-Department of Molecular Genetics, University of Antwerp, Campus Drie Eiken, Universiteitsplein 1, 2610 Antwerp, Belgium; Department of Neurology, Antwerp University Hospital, Antwerp, Belgium; Laboratories of Neurogenetics and Neuropathology, Institute Born-Bunge, University of Antwerp, Antwerp, Belgium; Department of Genetics, University of Groningen, University Medical Center Groningen, Groningen, The Netherlands; Department of Neurology, Donders Institute of Brain, Cognition and Behaviour, Radboud University Medical Center, Nijmegen, The Netherlands; Department of Human Genetics and Hussman Institute for Human Genomics, Miller School of Medicine, University of Miami, Miami, USA; Hertie-Institute for Clinical Brain Research, Department of Neurodegenerative Diseases, University of Tübingen, Tübingen, Germany; German Research Center for Neurodegenerative Diseases, Tübingen, Germany; Pediatrics Outpatient Clinic, Edegem, Antwerp, Belgium; University of Antwerp, Antwerp, Belgium

**Keywords:** Early onset cerebellar ataxia, Epilepsy, Intellectual disability, *KCND3*, SCA19/22, Channelopathy, Immunocytochemistry, Immunoblotting, Patch clamp study, Whole exome sequencing/WES

## Abstract

**Background:**

Identification of the first *de novo* mutation in *potassium voltage-gated channel, shal-related subfamily, member 3* (*KCND3*) in a patient with complex early onset cerebellar ataxia in order to expand the genetic and phenotypic spectrum.

**Methods:**

Whole exome sequencing in a cerebellar ataxia patient and subsequent immunocytochemistry, immunoblotting and patch clamp assays of the channel were performed.

**Results:**

A *de novo KCND3* mutation (c.877_885dupCGCGTCTTC; p.Arg293_Phe295dup) was found duplicating the RVF motif and thereby adding an extra positive charge to voltage-gated potassium 4.3 (Kv4.3) in the voltage-sensor domain causing a severe shift of the voltage-dependence gating to more depolarized voltages. The patient displayed a severe phenotype with early onset cerebellar ataxia complicated by intellectual disability, epilepsy, attention deficit hyperactivity disorder, strabismus, oral apraxia and joint hyperlaxity.

**Conclusions:**

We identified a *de novo KCND3* mutation causing the most marked change in Kv4.3’s channel properties reported so far, which correlated with a severe and unique spinocerebellar ataxia (SCA) type 19/22 disease phenotype.

## Background

The autosomal dominant spinocerebellar ataxias (AD SCAs) are neurodegenerative disorders causing progressive ataxia, dysarthria and eye movement difficulties [1]. Currently, more than 40 loci underlie the AD SCAs, including repeat expansions, rearrangements and point mutations [2, 3]. Recently, loss-of function mutations in *KCND3* (potassium voltage-gated channel, Shal-related subfamily, member 3) have been identified causing SCA19/22 [4, 5], whereas gain-of function mutations in *KCND3* were implicated in Brugada syndrome and atrial fibrillation [6–8]. *KCND3* encodes the voltage-gated potassium channel Kv4.3, a membrane protein that consists of six trans-membrane segments (S1-S6) and two intracellular tails. Four Kv4.3 subunits co-assemble to form the pore domain (helices S5-S6), the potassium selective conduction pathway. The S1-S4 segments form a single voltage-sensor domain that surrounds the pore domain, connected by the S4-S5 loop, and responds to changes in membrane voltages controlling the pore gates [9]. Kv4.3 rapidly activates and inactivates in response to membrane depolarization, contributing to the neuronal subthreshold A-type potassium currents and controlling the action potential repolarization and frequency, and thus neuronal excitability [10, 11]. The channel characteristics of Kv4.3 including protein trafficking, channel expression and activity can be modified by Kv channel-interacting protein 2 (KChIP) [12]. Eight different mutations in 10 SCA19/22 families have been published to date [4, 5, 8]. We expand the genetic and phenotypic spectrum by the identification of a *de novo* mutation in a patient with a severe and early onset phenotype.

## Methods

### Study participant

The index patient, a boy with early onset cerebellar ataxia, was investigated by a team of specialists in rare neurogenic disorders in the University Hospital of Antwerp (KS, BC, NVdA), laboratory serum tests for biomarkers and uric acid analysis were performed. Mutations in common ataxia genes were tested. Additionally, the boy underwent neuropsychological testing, nerve conduction studies, extensive cardiac evaluation and routine brain magnetic resonance imaging (MRI).

### Consent

Written informed consent was obtained from the parents and their five children for the publication of this report and any accompanying images. The Ethics Committee of Antwerp University approved this study.

### Whole exome sequencing

Genomic DNA was obtained from patient, parents and sibs. Exome capturing via the SureSelect, Human All Exon5 kit (50 Mb) (Agilent) and sequencing on a Hiseq 2000 sequencer (Illumina) was performed on the DNA of the patient. The Burrows-Wheeler Algorithm was used to align 100 bp length paired-end reads to the hg19 version of the human Genome (Ensembl). Variants were called with the Genome Analysis Toolkit (GATK) software package and data were imported into the Genomes Management Application (GEM-app) database [13]. A total of 104.3 million reads were produced for this sample, 97.9 % of which could be aligned to the targeted sequence. Mean coverage of the targeted sequence was 91-fold; 90322 single-nucleotide variations (SNV) and 9323 indels were called. Variants were filtered for occurrence in the normal population (minor allele frequency < 1 % in dbSNP138 and the Exome Variant Server), conservation (Genomic Evolutionary Rate Profiling [GERP] score > 4 or PhastCons score > 0.9), quality (GATK and Genotype Quality score > 75) and predicted impact on the encoded protein according to the refGene annotation (missense, nonsense, frame shift, inframe indels and essential splice variants). The remaining set of variants were filtered for a list of known ataxia genes, resulting in this unique *KCND3* variant.

This variant and its segregation was confirmed with Sanger sequencing in the index patient, his parents and four unaffected brothers. To proof the *de novo* character, paternity was tested using 15 highly informative STR (short tandem repeat) markers, distributed throughout the genome. STRs were PCR-amplified and polymerase chain reaction (PCR) fragments were loaded on an ABI 3730 automated DNA sequencer. Genotypes were analysed using the ABI Prism Genescan software (Applied Biosystems, Foster City, USA) and Trace Inspector, an in-house developed software program (http://www.vibgeneticservicefacility.be/).

### Plasmids

The duplication of the nucleotides 877_885 (CGCGTCTTC) in the human Kv4.3 cDNA was performed using site-directed mutagenesis (forward primer: GGTCTTCCGCGTCTTCCGCGTCTTCAGGATCTTCAAGTTTTC; reverse primer: GAAAACTTGAAGATCCTGAAGACGCGGAAGACGCGGAAGACC) and then subcloned into pcDNA3.1 as described previously [4]. The Emerald-KChIP2b was kindly provided by Dr. K. Takimoto (Nagaoka University of technology, Kamitomoika, Japan).

### Immunocytochemistry and immunoblotting

HeLa cells were transfected with pcDNA3.1-Kv4.3 wild type (WT) or duplicationRVF (dupRVF) mutant with or without Emerald-KChIP2 using polyethylenimine (Polysciences), according to the manufacturer’s instructions. Immunological techniques were performed as described previously [4, 8]. Anti-Kv4.3 (C-17; Santa Cruz) and anti-Golgin 97 (CDFX; Santa Cruz) antibodies were used for immunostaining, and anti-Kv4.3 (C-17; Santa Cruz), anti-KChIP2 (Abcam) and anti-actin (MP Biochemicals) antibodies for Western blot. Protein densitometry was quantified using the Quantity One program (Bio-Rad) and plotted. Data are represented as mean ± SEM (standard error of the mean) and the significance was calculated using Student’s *t*-test (*p < 0.01). Images were obtained by a DMI 6000 Inverted microscope (Leica) and processed using ImageJ software (National Institutes of Health).

### Electrophysiology

CHO-K1 cells (Chinese hamster ovary) were co-transfected with plasmids containing Kv4.3 WT or Kv4.3 dupRVF and Emerald-KChIP2 in a 1:1 ratio. Whole-cell patch clamp configuration was used to measure potassium currents as previously described with some modifications [4]. Briefly, potassium current recordings were evoked by depolarizing voltage steps (11 mV increments, 200 ms) ranging from −90 mV to potentials between −82 and +83 mV; current densities were plotted against the voltages. To compare the steady state properties of activation in WT and dupRVF channels, conductance was calculated from peak current amplitudes using reversal potential determined for each experiment and normalized to the maximum conductance value. Normalized conductance was plotted against the voltage. To measure the voltage-dependence of Kv4.3 steady-state inactivation, currents were obtained with a double pulse protocol in which a 200 ms conditioning pulse to potentials between −100 and +100 mV was applied from a holding potential of −90 mV and the steady-state inactivation was assessed from the peak outward current during the subsequent step to +60 mV (80 ms). Maximum currents of the second pulse were normalized against the maximum current of the first pulse. Normalized inactivation values were plotted against the voltages. Activation and inactivation curves were fitted in a single Boltzmann equation to obtain the half-maximum activation and inactivation voltages (V_1/2_) and slope factors. Data are presented as mean ± SEM (Standard Error of the Mean). Statistical analysis was perfume using Student’s *t*-test.

## Results

### Clinical assessment

**Fig. 1:**
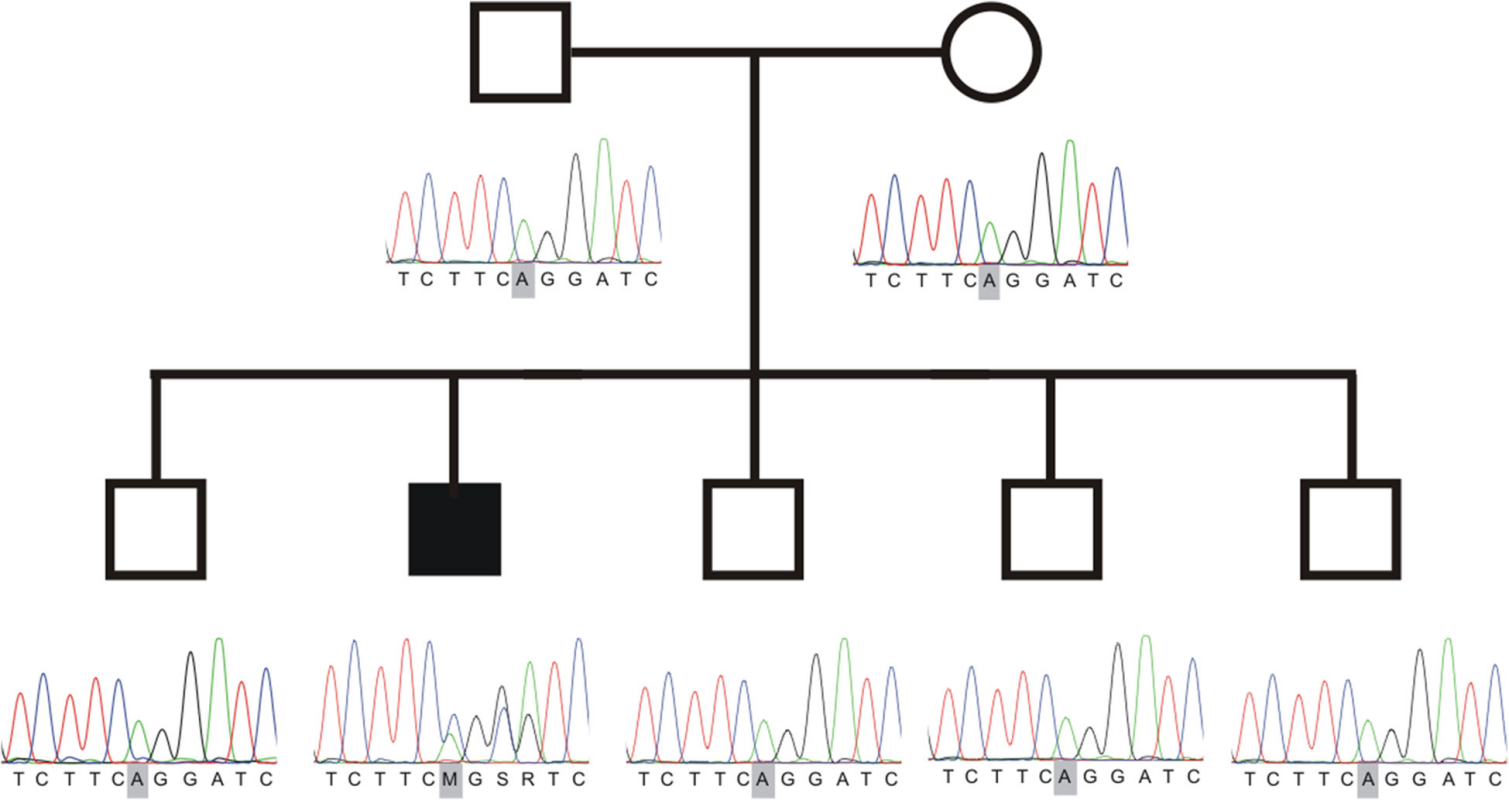
Pedigree and electropherogram of *de novo KCND3* mutation. Pedigree of the Belgian family. Squares indicate males and circle represent female. Filled symbol means affected individual. The affected boy carries the p.Arg293_Phe295dup mutation which is absent in the parents and four healthy brothers

The patient was born a term after an uncomplicated pregnancy and delivery. Both parents, non-consanguineous, of Belgian origin, and four male sibs were unaffected by history (Fig. 1, Pedigree). At the age of 3 years, clear slowing of motor milestones was noted with a progressive broad-based gait, staccato speech and intellectual disability (ID). He underwent surgery for strabismus at 4 years of age. Frequent nocturnal muscle jerks, episodes of staring and severe concentration problems occurred one year later. Attention deficit hyperactivity disorder (ADHD) was diagnosed but methylphenydate did not improve symptoms. Electroencephalograms showed frequent paroxysmal rhythmic theta waves in frontal and parietal regions. A diagnosis of generalized epilepsy was made and treatment with valproate was initiated alleviating the seizures. Treatment was stopped successfully at 9 years of age. At the age of 10 years, clinical examination showed a severe cerebellar atactic gait, severe cerebellar limb ataxia, a clear cerebellar dysarthria and saccadic eye movements. Reflexes and sensory examination were normal. Continuous hypersalivation due to oral apraxia was seen and a daily treatment with an anticholinergic drug, oral glycopyrronium was started. Joint hyperlaxity was seen as well.

### Auxiliary investigations

Neuropsychological testing at the age of 6 years showed a total IQ of 54 (verbal IQ 60, performal IQ 52). He was diagnosed with mild ID, normal education was impossible and he went to a special school. He was unable to write and to read, he was unable to ride his bike and used a tricycle. He needed daily physiotherapy, logopaedic training and occupational therapy. A brain MRI was performed, at the ages 6 and 10 years and was normal. Nerve conduction studies, electromyography, cardiac ultrasound, holter monitoring and ajmaline-testing at the age of 10 years were unremarkable.

### Serum Biomarkers and genetic analyses

The index patient was also assessed by extensive serum testing for biomarkers indicative of recessive ataxias (alpha-fetoprotein, lactate, vitamin E, very long fatty acids, phytanic acid, coeruloplasmin, cholesteanol, lysosomal enzymes, quantitative assessment of amino acids) [14], and genetic testing for SCA1,2,3,6,7 was performed. All results were normal.

### Whole exome sequencing

**Fig. 2:**
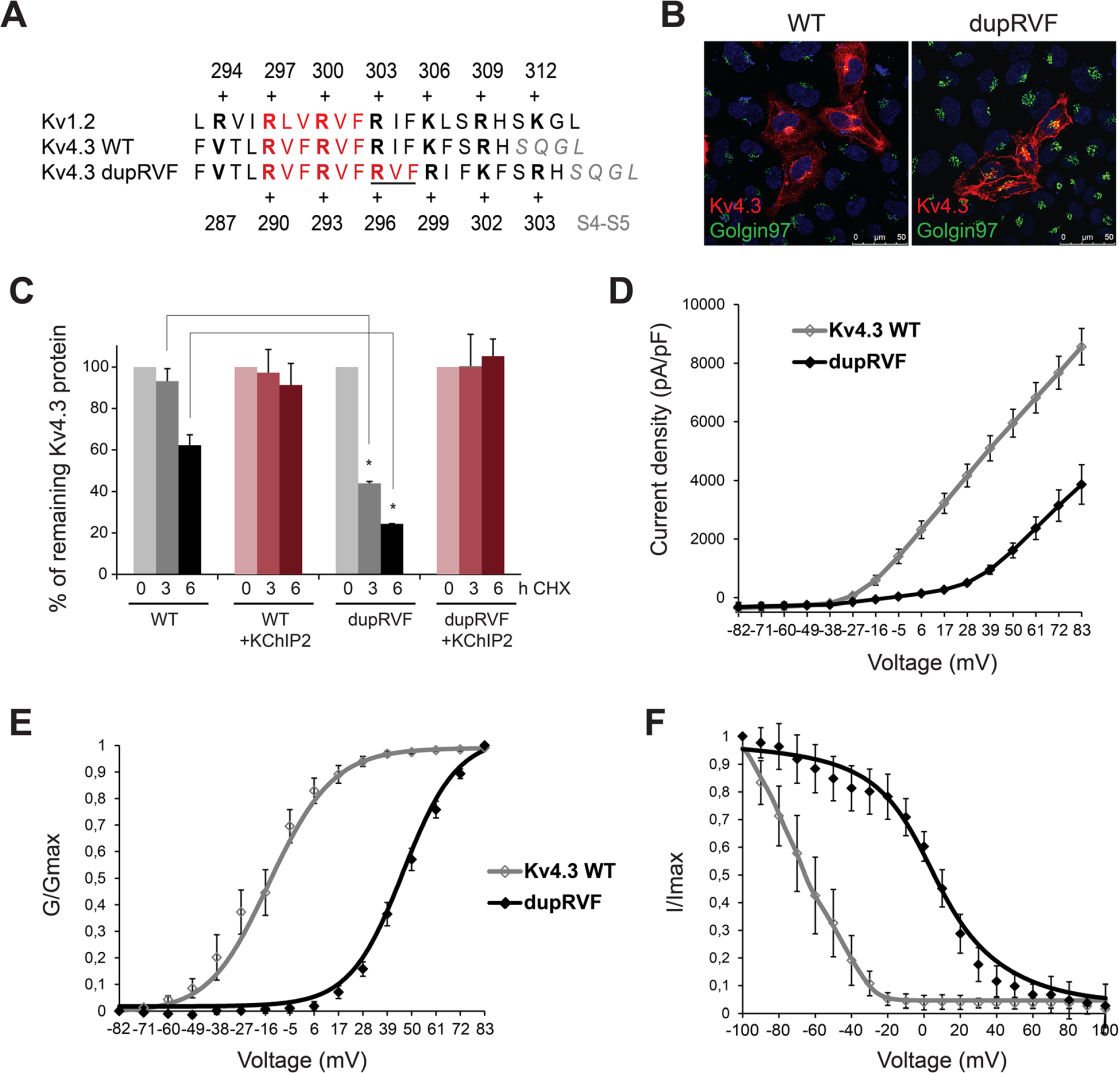
Biochemical and functional analysis of Kv4.3 dupRVF mutant channel. **a** Sequence comparison of the S4 transmembrane segment for Kv1.2 and Kv4.3 WT and dupRVF mutant channels. Insertion of RVF at amino acid position 296–298 is underlined. **b** Co-immunostaining of Kv4.3 WT or dupRVF mutant (red) and golgin97 (Golgi apparatus marker; green) in transfected HeLa cells. Nuclei were stained using Dapi (blue). Scale bar = 50 μm. **c** Percentage of remaining Kv4.3 protein after 0, 3 h or 6 h cycloheximide (CHX) treatment. In absence of KChIP, the remaining dupRVF mutant protein was significantly lower compare with the WT at 3 h (dupRVF: 43.9 ± 0.9 % *vs* WT: 93.1 ± 6 %) and at 6 h (dupRVF: 24.4 % *vs* WT: 62.2 ± 5 %). In presence of KChIP, no significant differences were detected at 3 h (dupRVF: 100.4 ± 15 % *vs* WT: 97.2 ± 11 %) and at 6 h (dupRVF: 105.2 ± 8 % *vs* WT: 91.3 ± 10 %). Graph represents the Western blot densitometries and is representative of 3 independent experiments. Significant differences are depicted by the Student’s *t* test (*p* > 0.01). **d** Averaged current density-voltage relationships of Kv4.3 WT and dupRVF mutant, expressed in CHO cells with KChIP2 (ratio 1:1). **e** Normalized conductance-voltage relationship for Kv4.3 WT and dupRVF mutant. **f** Voltage-dependent inactivation curves of Kv4.3 WT and dupRVF. Data were fitted to Boltzmann function (solid curves) and parameters summarized in Table 1

Subsequently whole exome sequencing (WES) was peformed and this revealed a *KCND3* mutation (c.877_885dupCGCGTCTTC; p.Arg293_Phe295dup), (RefSeq NM_004980.4), resulting in the duplication of the RVF (Arginine-Valine-Phenylalanine) motif (Fig. 2a). The mutation was confirmed to be *de novo* by Sanger sequencing. The four healthy brothers did not carry the mutation (Fig. 1, pedigree).

### Immunocytochemistry, immunoblotting and patch clamp studies

To test whether the duplication of the RVF motif in the S4 segment of Kv4.3 (Fig. 2a) alters the intracellular trafficking and the stability of the channel we performed immunocytochemistry and time-course cycloheximide (CHX) experiments on fixed HeLa cells expressing Kv4.3 WT or dupRVF mutant. Kv4.3 harboring the dupRVF was mainly detected at the cell surface similar to the WT, as was shown by confocal microscopy (Fig. 2b). Despite proper cellular localization, dupRVF mutant Kv4.3 showed significant decreased protein stability compared with WT after 6 h of CHX treatment (Fig. 2c; grey-black bars). KChIP2 could rescue the protein instability of dupRVF mutant Kv4.3 (Fig. 2c; pink bars).

To determine the effect of the dupRVF mutations on channel activity, we measured the currents of Kv4.3 WT and dupRVF in the presence of KChIP2 (1:1) in CHO cells. The average peak outward current at +83 mV for dupRVF was strongly reduced (55 %) compared to WT (Fig. 2d; Table 1). Additionally, the dupRVF induced a dramatic depolarizing shift in the voltage dependence of Kv4.3 activation of about +59.3 mV, with no changes in the slope factor (Fig. 2e, Table 1), and a dramatic depolarizing shift in the voltage dependence of inactivation of about +62 mV, with an increased slope factor (Fig. 2f; Table 1).

**Table 1:** Summary of Kv4.3 WT and dupRVF mutant channel properties

Kv4.3	Current density at +83 mV (pA)	Activation		Inactivation	
		V_1/2_ (mV)	Slope (mV)	V_1/2_ (mV)	Slope (mV)
WT (*n* = 8)	8561 ± 620	−13.3 ± 3.2	12.7 ± 0.8	−59.7 ± 6.1	4.7 ± 0.5
dupRVF (*n* = 9)	3861 ± 668**	46.0 ± 2.1**	11.7 ± 0.5	2.3 ± 7.0**	15.3 ± 2.0*

Values are means ± SEM

Statistical analysis *t*-test: *, *p* < 0.001; **, *p* < 0.0001

These data show that the duplication of the RVF motif in the voltage-sensor of Kv4.3 does not affect channel trafficking to the plasma membrane but does cause protein instability that could be corrected with the regulatory KChIP2. This mutation caused a strong shift in the voltage-dependence of activation and inactivation that very likely explains the disease symptoms.

## Discussion

Mutations in *KCND3* are an uncommon cause of cerebellar ataxia [4, 5]. Eight different *KCND3* mutations have been described in one large Dutch family [4], one large Chinese family [5] and in eight smaller Dutch, Ashkenazi-Jewish American, Japanese and French families [4, 8]. We describe the first *de novo KCND3* mutation in a Belgian patient. Our patient has an unusually severe and complex phenotype with onset at the age of 3 years, the youngest onset reported so far. The previously described SCA19/22 families had disease-onset at 10–55 years of age and presented with milder cerebellar ataxia [4, 5]; even in those with earlier onset of disease, the progression of cerebellar ataxia was slower compared to our case. Our patient exhibited mild intellectual disability (ID) and ADHD; while moderately reduced IQ has been described in one of the Dutch families, ADHD was not reported [15]. Neither cognitive impairment nor behavioural dysfunction were noticed in the large Chinese *KCND3* kindred [5]. Our patient also had mild generalized seizures responding well to valproate. Epilepsy has previously not been linked to SCA19/22, although two Dutch patients had clinical and electrophysiological findings indicative of cortical (and spinal) myoclonus [15]. Genes encoding potassium channels have been frequently reported in epilepsy and ataxia syndromes, including *KCNQ2/KCNQ3* in benign familial neonatal convulsion syndromes [16, 17], *KCNCI* in progressive myoclonus epilepsy [18], *KCNQ2* in epileptic encephalopathy syndromes [19], *KCNA1* in episodic ataxia type 1 [20], *KCNC3* in SCA13 [21] and recently myoclonic epilepsy and ataxia in *KCNA2* [22]. Other unique phenotypic features in our case were strabismus, severe oral apraxia, and joint hyperlaxity, which are all supposedly part of the phenotype of this de novo *KCND3* mutation.

In our patient, we did not observe the cerebellar vermis atrophy that was documented in previous SCA19/22 cases [15]. The patient’s young age and relatively short disease duration might account for this.

Cardiac evaluation was normal in our patient, in particular there were no cardiac arrhythmias or other conduction abnormalities. The duplication of codons 293–295 does not target the S6 and C terminal tail, where most Brugada syndrome or atrial fibrillation mutations reside [6, 7]. Some mutations may cause either ataxia or Brugada syndrome/atrial fibrillation, however, the co-occurrence of both diseases has not been reported [8].

The insertion of the RVF motif at amino acid position 296–298 in Kv4.3 introduces an extra arginine in the S4 segment resulting in a more positively charged alpha helix S4 (Fig. 2a). Changes in the conformation of the voltage-sensor domain (S1-S4) may alter the voltage-dependent gating properties of the Kv4.3 channel [23, 24]. No trafficking deficit was observed for the dupRVF mutant suggesting that the RVF duplication does not markedly alter the S4 domain alpha-helix structure, but does cause protein instability. However, this mutation caused a strong shift in the voltage-dependence of activation and inactivation of Kv4.3. The alteration in channel gating observed for dupRVF mutant may underlie the neuronal death due to marked changes in neuronal excitability. Moreover, the protein instability of dupRVF mutant suggests that haploinsufficiency could also contribute to the disease pathology.

From all the described and characterized Kv4.3 mutations, the p.F227del causes similar alterations in Kv4.3 channel functioning as the p.Arg293_Phe295dup [5, 25]. However, the effects of p.F227del on channel gating are minor compared to p.Arg293_Phe295dup, as p.F227del led to activation (+28 V) and inactivation (+8 V) shifts to more positive voltages in oocytes [25]. These relatively minor changes in channel gating are reflected in the late age of onset and mild SCA19/22 disease symptoms [5].

Previous work showed that replacing positively charged arginines in S4 of Kv4.3 by non-charged amino acids, shifted activation and inactivation of Kv4.3 to more negative voltages [23]. This links SCA19/22 to other neurological potassium channelopathies where an alteration of the gating properties of voltage-gated potassium channels is caused by alterations of positive residues in their voltage-sensor domains [19], e.g. *KCNQ2* in peripheral nerve hyperexcitability with myokymia [26], *KCNQ2/KCNQ3* in benign familial neonatal convulsions [16, 17, 27] and *KCNQ2* in epileptic encephalopathy [19].

## Conclusions

While most SCA subtypes are inherited in a clear autosomal dominant fashion, our case adds evidence that *de novo* inheritance must be increasingly taken into consideration when analyzing ataxia pedigrees and SCA exomes. This is also of particular importance given the fact that the phenotype of our case - severe early onset ataxia with multisystemic neurological damage and lack of neurological disease in the parental generation - closely resembles the phenotype known from many other recessive ataxias, thus mimicking autosomal recessive inheritance.

To our knowledge, this *de novo* mutation causes the most marked change in Kv4.3’s channel properties reported so far and leads to a uniquely severe early onset SCA19/22 phenotype.

## Abbreviations

AD: Autosomal dominant
ADHD: Attention deficit hyperactivity disorder
CHO: Chinese hamster ovary
CHX: Cycloheximide
ID: Intellectual disability
*KCND3*: Potassium voltage-gated channel, shal-related subfamily, member 3
KChIP2: Voltage-gated potassium channel-interacting protein 2
Kv: Voltage-gated potassium
RVF: Arginine-Valine-Phenylalanine
dupRVF: DuplicationRVF
SCA: Spinocerebellar ataxia
SEM: Standard error of the mean
WES: Whole exome sequencing
WT: Wild type
